# Coupled Node Similarity Learning for Community Detection in Attributed Networks

**DOI:** 10.3390/e20060471

**Published:** 2018-06-17

**Authors:** Fanrong Meng, Xiaobin Rui, Zhixiao Wang, Yan Xing, Longbing Cao

**Affiliations:** 1 School of Computer Science and Technology, China University of Mining and Technology, Xuzhou 221116, China; 2 School of Computer Science and Technology, Civil Aviation University of China, Tianjin 300300, China; 3 Advanced Analytical Institute, University of Technology Sydney, Sydney, NSW 2007, Australia

**Keywords:** attributed networks, coupled node similarity, community detection

## Abstract

Attributed networks consist of not only a network structure but also node attributes. Most existing community detection algorithms only focus on network structures and ignore node attributes, which are also important. Although some algorithms using both node attributes and network structure information have been proposed in recent years, the complex hierarchical coupling relationships within and between attributes, nodes and network structure have not been considered. Such hierarchical couplings are driving factors in community formation. This paper introduces a novel coupled node similarity (CNS) to involve and learn attribute and structure couplings and compute the similarity within and between nodes with categorical attributes in a network. CNS learns and integrates the frequency-based intra-attribute coupled similarity within an attribute, the co-occurrence-based inter-attribute coupled similarity between attributes, and coupled attribute-to-structure similarity based on the homophily property. CNS is then used to generate the weights of edges and transfer a plain graph to a weighted graph. Clustering algorithms detect community structures that are topologically well-connected and semantically coherent on the weighted graphs. Extensive experiments verify the effectiveness of CNS-based community detection algorithms on several data sets by comparing with the state-of-the-art node similarity measures, whether they involve node attribute information and hierarchical interactions, and on various levels of network structure complexity.

## 1. Introduction

Community detection is an important task in complex network analysis. So far, the definition of community is still ambiguous. In most state-of-the-art research, the concept of community is a group of nodes densely connected relatively to the rest of the network. Networks that consider both object interactions and attributes, i.e., *attributed networks*, can be represented by an attributed graph in which nodes represent the objects, edges represent the relationships between objects, and the feature vectors associated with nodes represent the attributes. The network topological structure reflects the interactions between nodes and the node attribute information reflects the common characteristics among nodes. They both play important roles in the formation of the network community structure. However, nowadays most community detection algorithms only use the network topological structure. Community detection on such attributed networks using both network topological structure and node attribute information is important yet challenging, and relies on appropriate similarity learning.

**Community detection on attributed networks.** Nowadays, many approaches have been proposed that incorporate node attributes and edges in the community detection process. Existing methods can be classified roughly into two categories. The first category is composed of probabilistic generative models that formulate joint models of edges and node attributes, and that use the models to infer the community memberships of nodes in an attributed network [1,2,3,4]. However, they are not as efficient as hybrid methods. Cruz et al. [5] proposed an iterative optimization algorithm by maximizing the modularity and entropy to obtain the structural and semantically related community structures. CODICIL [6] constructs content edges by selecting the top *K* neighbors of each node using their attributes, obtains the combined similarity of each pair of nodes, and then sparsifies the newly constructed graph with content edges. Finally, an existing community detection algorithm is used to partition the sparsified graph into a given number of communities. SA-cluster [7] views node attributes as virtual vertices, constructs an attribute-augmented graph, and performs a random walk on the attribute-augmented graph to obtain a unified distance. It then adopts the K-medoids algorithm to detect the community based on learned pairwise distance. Inc-cluser [8,9] is a slightly faster version of SA-cluster.

**Related work on similarity learning in community detection.** In understanding attributed graphs, many methods take the following strategy. First, an attributed graph is converted (reduced) to a weighted graph, where weights represent attribute similarity. The edge weights indicate particularly close connections or similarity between nodes. Then, clustering algorithms for weighted graphs can be applied. For example, Steinhaeuser and Chawla [10] presented a simple approach to constructing a network with edge weights based on node attributes and clustering nodes whose edge weight exceeds the threshold in the same community. The authors show that edge weights based on node attribute similarity are superior to edge weights based on network topology in a large scale-free social network.

Appropriately, learning node similarity is critical for understanding network complexity and effectively detecting communities. Accordingly, a number of methods have been developed that exploit node similarity by capturing node relationships. Node similarity learning in existing methods can be roughly assigned to two categories: *structure similarity* and *attribute similarity*. Structure similarity, which focuses on so-called structure equivalence, is commonly used; that is, two nodes are similar if they share the same or similar network neighbors. Typical examples include the cosine similarity (Cosine) [11] and the Jaccard index (Jaccard) [12]. The other kind of methods collect both local and global scale information to compute the weight of each edge, including k-path edge centrality [13], SimRank-based edge weighting scheme [14], and WNF [15]. Few attribute similarity learning methods are proposed for categorical network data. The representative methods are simple matching coefficient (SMC) [16] and the recently proposed coupled object similarity (COS) [17,18]. COS has been shown to be effective in categorical data analysis for clustering [19], classification [20], and recommender systems [21], as it aims to capture the value-to-object coupling relationships [22] embedded in a complex dataset.

To the best of our knowledge, there are no such node similarity learning methods in attributed network analysis that effectively capture the complex interactions between node attributes and network structure, and the coupling relationships within and between node attributes. These complex interactions and relationships drive the formation of communities, so it is fundamental to understand how they interact and affect community and network dynamics.

**Figure 1:**
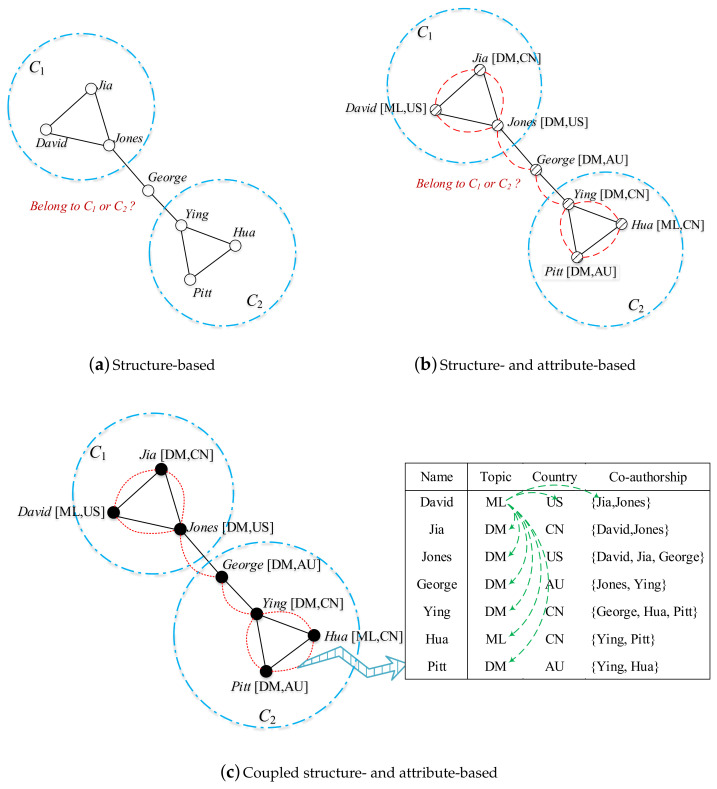
Structure and attribute couplings in a co-authoring network. (Note: Symbol 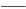 indicates the linkage between two nodes; 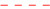 refers to simple attribute similarity between two nodes, and 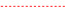 represents the complex coupling relationships between two nodes.)

**Learning complex coupling relationships and our main contributions.** We illustrate the various coupling relationships in a co-authoring network in Figure 1, in which the information table shows the co-authoring information. A node represents an author, and an edge represents the co-authoring relationship between two authors. In addition, there is topic and country information associated with each author. We convert the co-authoring information table to graphs based on different approaches to represent the structure and relationships between authors. Below, we discuss the different outcomes of author communities detected as a result of these different representation approaches.

First, if only the structure of the co-authoring graph is considered in the detection of author communities, we have the co-authoring structure shown in Figure 1a. Two clearly separated communities emerge: {David, Jia, Jones} and {Ying, Hua, Pitt}; however, we cannot tell which community George belongs to since he has the same relationship with Jones and Ying who separately belong to two different communities. Second, if both graph structure and node attributes are considered, which results in the diagram in Figure 1b, we still cannot cluster George to a proper community by using SMC to compute the similarity between two connected authors. Since SMC uses 0 and 1 to distinguish the similarity between categorical values, the similarity between authors who live in AU and the US is equal to that between authors who live in AU and CN. Therefore, the similarity between George and Jones is still same as the similarity between George and Ying. However, by involving the co-authoring relationships in Figure 1c, we observe that the similarity between AU and CN should be greater because authors from these two countries collaborate more frequently. Therefore, George is more similar to Ying than Jones, and we can correctly divide George to the right community.

The above three scenarios illustrate the importance of involving relevant information and relationships and learning their similarity in community detection. As shown in the limited work reported in the literature [23], engaging both structure and attribute similarities can generate more meaningful communities. However, existing methods do not consider the complex interactions within and between attributes, and between node attributes and structure. In most attributed networks, nodes prefer to connect to other nodes with similar attributes (i.e., homophily) [24]. The presence of homophily has been discovered in a vast array of network studies. More than 100 studies that have observed homophily in some form or another and they establish that similarity breeds connection [25]. The homophily property reflects the effect of node attributes on the network edges. On the other hand, the edges in the network should also reflect the difference between attributes. In this paper, we propose a novel coupled node similarity (CNS) learning method, which involves both node attributes and structure information in an attributed graph. The main idea behind CNS and its contributions to community detection are presented below:

- CNS captures different levels of coupling relationships in an attributed graph, including value-to-value, value-to-node, and attribute-to-structure relationships. To the best of our knowledge, this is the first work that systematically represents the hierarchical interactions in terms of both structural and attribute aspects.
- CNS learns the above respective relationships in terms of calculating and integrating the intra-attribute coupled similarity, the inter-attribute coupled similarity, and the coupled attribute-to-structure similarity. Hence, CNS captures not only the attribute value interactions within and between attributes, but also the interactions between node attributes and structure. This provides a comprehensive means of understanding the intrinsic driving forces and complexity in community formation.
- We incorporate CNS into attributed graphs to generate weighted graphs, combining the topological structure and node attributes in a unified manner to detect communities in attributed networks.
- We also empirically evaluate the effectiveness of CNS similarity in terms of whether node attributes are involved, what types of node interactions are learned, and different levels of network structure complexity.

## 2. Learning Coupled Node Similarity

In this section, we introduce the framework and specific similarity measures for learning coupled node similarity.

### 2.1. The CNS Framework

**Figure 2:**
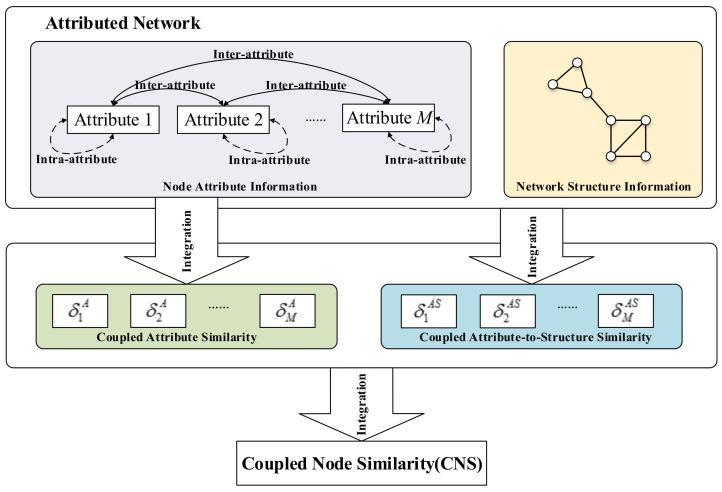
The framework for learning CNS. (Note: Symbol ⤎⤏ indicates intra-attribute coupled similarity calculated using the interaction between attribute values within an attribute and ⟷ refers to inter-attribute coupled similarity involved the couplings between attributes. The coupled attribute similarity in the second level integrates both of intra-attribute coupled similarity and inter-attribute coupled similarity. The coupled attribute-to-structure similarity in the second level captures the interactions between node attributes and network structure. In the last level, CNS integrates the coupled attribute similarity and the coupled attribute-to-structure similarity.)

The framework for learning CNS is shown in Figure 2. CNS captures four sources of interactions and similarities: (1) the *intra-attribute coupled similarity* learns the interactions within a node attribute; (2) the *inter-attribute coupled similarity* models the interactions between node attributes; (3) the *coupled attribute similarity* integrates both of them; and (4) the *coupled attribute-to-structure similarity* captures the interactions between node attributes and network structure. Lastly, CNS integrates the coupled attribute similarity and the coupled attribute-to-structure similarity to represent the overall relationships and similarities in an attributed network.

An attributed network can be modeled as a graph G=(V,E,F), where *V* is the set of nodes, *E* is the set of edges, and *F* is the set of node attribute vectors. All the main notations are described in Table 1.

### 2.2. Coupled Attribute Similarity

Coupled attribute similarity (CAS) is extended from the concept of Coupled Attribute Similarity for Object (CASO) in Wang et al. [18]. CASO is based on the coupled attribute similarity for values, by considering both the intra-coupled and inter-coupled attribute value similarities, which globally capture the attribute value frequency distribution and attribute dependency aggregation with high accuracy and relatively low complexity. CAS combines the intra-attribute coupled similarity (Defintion 1) and inter-attribute coupled similarity (Defintion 2) to cater for specific characteristics in network data.

**Definition** **1.** *(Intra-Attribute Coupled Similarity) The intra-attribute coupled similarity $\delta_{m}^{Ia}\left(x,y\right)$ between node attribute values where x and y, x= $F_{m}\left(i\right)$ and $y=F_{m}\left(j\right)$ are the values of nodes i and j in the mth attribute, is calculated by considering the relationship between the frequency of their occurrence.*

(1) $$\delta_{m}^{Ia}\left(x,y\right)=\frac{|g_{m}\left(x\right)|\times |g_{m}\left(y\right)|}{|g_{m}\left(x\right)|+|g_{m}\left(y\right)|+|g_{m}\left(x\right)|\times |g_{m}\left(y\right)|}$$

*$g_{m}\left(x\right)$ and $g_{m}\left(y\right)$ are the node sets which have the same attribute value as nodes i and j, respectively, in the mth attribute. $|g_{m}\left(x\right)|$ and $|g_{m}\left(y\right)|$ are the occurrence times of node attribute values x and y across all nodes in the network.*

In the toy example in Figure 1, for example, there are two authors from Australia {George, Pitt} and three from China {Ying, Hua, Jia}, so $\delta_{country}^{Ia}\left(AU,CN\right)=6/11$.

Below, the *inter-attribute coupled similarity* is defined, which considers the couplings between node attributes when the node attribute value similarity is calculated.

**Definition** **2.** *(Inter-Attribute Coupled Similarity) The inter-attribute coupled similarity $\delta_{m}^{Ie}\left(x,y\right)$ between values x and y of the mth attribute based on other attributes is defined as follows.*

(2) $$\delta_{m}^{Ie}\left(x,y\right)=\sum_{n=1,n\neq m}^{M}\alpha_{n}\delta_{m|n}\left(x,y\right).$$

*$\alpha_{n}$ is the weight parameter for the nth attribute, $\sum_{n=1}^{M}\alpha_{n}=1$, $\alpha_{n}\in \left[0,1\right]$. M is the total number of node attributes. $\delta_{m|n}\left(x,y\right)$ is one of the inter-relative attribute coupled similarities between values x and y of the mth attribute based on the nth attribute (n≠m).*

(3) $$\delta_{m|n}\left(x,y\right)=\min_{B\subseteq F_{n}}\left\{2-P_{n|m}\left(B|x\right)-P_{n|m}\left(\bar{B}|y\right)\right\}.$$

*$F_{n}$ represents the attribute values on the nth attribute. B is a subset of attribute values on the nth attribute. $\bar{B}=F_{n}/B$ is the complement set of B under the complete distinct value set $F_{n}$ of the nth attribute. $P_{n|m}\left(B|x\right)$ is the information conditional probability (ICP) of B with respect to x, which is defined as follows.*

(4) $$P_{n|m}\left(B|x\right)=\frac{|g_{n}^{*}\left(B\right)\cap g_{m}\left(x\right)|}{|g_{m}\left(x\right)|}.$$

$g_{n}^{*}\left(B\right)$ is the node set whose attribute value in the nth attribute is in B. Intuitively, when given all the objects with the value x on mth attribute, ICP is the percentage of common objects whose values on the nth attribute fall in subset B and whose values on the mth attribute are exactly x as well.

In the toy example in Figure 1, $F_{topic}=\left\{DM,ML\right\}$, and the number of its power sets is four. If B={DM} then $\bar{B}=\left\{ML\right\}$, $g_{topic}^{*}\left(B\right)=\left\{Jia,Jones,George,Ying,Pitt\right\}$, $g_{country}\left(AU\right)=\left\{George,Pitt\right\}$, $P_{topic|country}\left(B|AU\right)=1$. Similarly, $P_{topic|country}\left(\bar{B}|CN\right)=1/3$. Considering all conditions, $\delta_{country|topic}\left(AU,CN\right)=2/3$. Since there are only two attributes, $\delta_{country}^{Ie}\left(AU,CN\right)=2/3$.

**Definition** **3.** *(Coupled Attribute Similarity) The coupled attribute similarity $\delta_{m}^{A}\left(x,y\right)$ between values x and y of the mth attribute is the combination of the intra-attribute coupled similarity and the inter-attribute coupled similarity between x and y.*

(5) $$\delta_{m}^{A}\left(x,y\right)=\delta_{m}^{Ia}\left(x,y\right)\times \delta_{m}^{Ie}\left(x,y\right).$$

Lastly, the *coupled attribute similarity (CAS)* for the two nodes *i* and *j* is calculated as follows.

(6) $$CAS\left(i,j\right)=\sum_{m=1}^{M}\delta_{m}^{A}\left(F_{m}\left(i\right),F_{m}\left(j\right)\right).$$

### 2.3. Coupled Attribute-to-Structure Similarity

In an attributed network, not all node attributes are equally important for community detection; even for an attribute, two different value pairs may not contribute the same. Based on the homophily property [24] of social networks, i.e., nodes to be connected with other nodes that share similar attributes, the consistency between node attributes and structure information could guide the community detection process. Therefore, the coupled attribute-to-structure similarity is proposed to measure the different contribution of different attribute value pairs.

**Definition** **4.** *(Coupled Attribute-to-Structure Similarity) The coupled attribute-to-structure similarity $\delta_{m}^{AS}\left(x,y\right)$ between values x and y of the mth attribute is defined as the degree of consistency between the attribute value pair (x,y) and the linkage across all nodes in the network. It is equal to the number of edges between the two node sets whose attribute values are x and y, respectively, in the mth attribute divided by the total number of possible edges between them.*

(7) $$\delta_{m}^{AS}\left(x,y\right)=\frac{\sum_{v_{1}\in g_{m}\left(x\right),v_{2}\in g_{m}\left(y\right)}A\left(v_{1},v_{2}\right)}{|g_{m}\left(x\right)|\times |g_{m}\left(y\right)|}.$$

In the toy example in Figure 1, there are two authors from Australia and three from China, and there are three connections between the authors from these two countries, {George-Ying, Pitt-Ying, Pitt-Hua}, so $\delta_{country}^{AS}\left(AU,CN\right)=0.5$.

### 2.4. Coupled Node Similarity

*Coupled node similarity* is defined as the combination of the coupled attribute similarity and coupled attribute-to-structure similarity.

**Definition** **5.** *(Coupled Node Similarity) The coupled node similarity CNS(i,j) between nodes i and j is calculated below:*

(8) $$\begin{array}{cc} CNS(i,j) & =\sum_{m=1}^{M}\delta_{m}^{A}\left(F_{m}\left(i\right),F_{m}\left(j\right)\right)\times \delta_{m}^{AS}\left(F_{m}\left(i\right),F_{m}\left(j\right)\right)\\ & =\sum_{m=1}^{M}\delta_{m}^{Ia}\left(F_{m}\left(i\right),F_{m}\left(j\right)\right)\times \delta_{m}^{Ie}\left(F_{m}\left(i\right),F_{m}\left(j\right)\right)\times \delta_{m}^{AS}\left(F_{m}\left(i\right),F_{m}\left(j\right)\right) \end{array}.$$

In the toy example in Figure 1, CNS(George,Ying)=0.41, CNS(George,Jones)=0.29, and George is more similar to Ying, so he belongs to community $C_{2}$.

### 2.5. The Algorithm for Learning CNS

Algorithm 1 presents the process of learning coupled node similarity (CNS). It first calculates the coupled attribute similarity and the coupled attribute-to-structure similarity for all attribute value pairs (Lines 1–8) and then computes CNS for all nodes (Lines 9–17). The CASS function computes the coupled attribute-to-structure similarity (Lines 19–24).
**Algorithm 1** Learning Coupled Node Similarity**Input:**G(V,E,F) **Output:**CNS1:**for**m←1,M**do**2:  **for all** value pairs x,y∈ unique $\left(F_{m}\right)$ **do**3:    $\delta_{m}^{Ia}\left(x,y\right)=CIAAS\left(x,y,m\right)$4:    $\delta_{m}^{Ie}\left(x,y\right)=CIEAS\left(x,y,m\right)$5:    $\delta_{m}^{A}\left(x,y\right)=\delta_{m}^{Ia}\left(x,y\right)\times \delta_{m}^{Ie}\left(x,y\right)$6:    $\delta_{m}^{AS}\left(x,y\right)=CASS\left(x,y,m\right)$7:  **end for**8:**end for**9:**for all** nodes *i* and *j* ∈V **do**10:  **for** m←1,M **do**11:    $x=F_{m}\left(i\right)$ , $y=F_{m}\left(j\right)$12:    **if** A(i,j)==1 **then**13:      $CNS\left(i,j\right)+=\delta_{m}^{A}\left(x,y\right)\times \delta_{m}^{AS}\left(x,y\right)$14:    **end if**15:  **end for**16:**end for**17:**return**CNS18: 19:**Function**CASS(x,y,m)20:**for all** nodes $v_{1}\in g_{m}\left(x\right)$ and $v_{2}\in g_{m}\left(y\right)$ **do**21:  $Enum+=A(v_{1},v_{2})$22:**end for**23:$\delta_{m}^{AS}\left(x,y\right)=Enum/\left(\right|g_{m}\left(x\right)|\times |g_{m}\left(y\right)\left|\right)$24:**return**$\delta_{m}^{AS}\left(x,y\right)$25: 26:**Function**CIAAS(x,y,m)27:$U_{1}\leftarrow \left\{v_{i}|F_{m}\left(i\right)==x\right\}$, $U_{2}\leftarrow \left\{v_{i}|F_{m}\left(i\right)==y\right\}$28:$\delta_{m}^{Ia}\left(x,y\right)=\left(\right|U_{1}\left|\times \right|U_{2}\left|\right)/\left(\right|U_{1}\left|+\right|U_{2}\left|+\right|U_{1}\left|\times \right|U_{2}\left|\right)$29:**return**$\delta_{m}^{Ia}\left(x,y\right)$30: 31:**Function**CIEAS(x,y,m)32:$U_{1}\leftarrow \left\{v_{i}|F_{m}\left(i\right)==x\right\}$, $U_{2}\leftarrow \left\{v_{i}|F_{m}\left(i\right)==y\right\}$33:**for**(n←1,M)and(n≠m)**do**34:  **for all** subset $B\in F_{m}$ **do**35:    $U_{3}\leftarrow \left\{v_{i}|F_{m}\left(i\right)\in B\right\}$, $U_{4}\leftarrow \{v_{i}|v_{i}\left|F_{m}\left(i\right)\in \left(F_{m}-B\right)\right\}$36:    $ICP_{x}\left(B\right)=\left(\right|U_{1}\left|⋂\right|U_{3}\left|\right)/\left(\right|U_{1}\left|\right)$37:    $ICP_{y}\left(F_{m}-B\right)=\left(\right|U_{2}\left|⋂\right|U_{4}\left|\right)/\left(\right|U_{2}\left|\right)$38:  **end for**39:  $Min_{m|n}=min\left(2-ICP_{x}-ICP_{y}\right)$40:  $\delta_{m}^{Ie}\left(x,y\right)+=\alpha_{n}\times Min_{m|n}$41:**end for**42:**return**$\delta_{m}^{Ie}\left(x,y\right)$

### 2.6. Complexity Analysis

CNS integrates three similarities, e.g., The intra-attribute coupled similarity, the inter-attribute similarity, and the coupled attribute-to-structure similarity. The time complexity analysis is as follows: (1) Compute intra-attribute coupled similarity: $O(MR^{2}|V|)$, where |V| is the number of nodes in the network; (2) Compute inter-attribute coupled similarity: $O(M^{2}R^{2}2^{R}|V|)$, where *R* is the maximal number of values for each attribute and *M* is the number of node attributes; (3) Compute coupled attribute-to-structure similarity: $O(MR^{2}|V|)$. Therefore, the overall time complexity is $O(M^{2}R^{2}2^{R}|V|)$.

## 3. Similarity-Based Community Detection

Our proposed method mainly concentrates on unweighted graphs. CNS is used to generate the edge weight (W(i,j)) where an edge exists when two nodes are linked structurally.

(9) $$W\left(i,j\right)=\left\{\begin{array}{cc} S(i,j) & \;if\;A(i,j)=1\\ 0 & otherwise \end{array}\right..$$

S(i,j) represents a similarity metric (e.g., CNS(i,j)) to be used to construct the weighted network. SLPA [26], BGLL [27], and *K*-medoids [28] then detect communities on the weighted networks.

SLPA is an extension of LPA [29] that can analyze communities in weighted networks. It starts by giving each node a unique label and provides each node with a memory to store received labels. In every iteration, each node receives labels from its neighbors and adds the most popular label to its memory. The most popular label is that which carries the maximum weight according to nodes that send the same label. Lastly, every node chooses the maximum frequent label in its memory as its community label and nodes with the same label are assigned to one community.

(10) $$l_{r}\left(i\right)=\underset{l}{\mathrm{argmax}}\sum_{j\in \Gamma_{i}}W\left(i,j\right)\times \varphi \left(l_{s}\left(j\right),l\right).$$

$l_{r}\left(i\right)$ represents the received label of node *i* and $l_{s}\left(j\right)$ is the send label from node *j*. If $l_{s}\left(j\right)=l$, then $\varphi (l_{s}\left(j\right),l)=1$, else $\varphi (l_{s}\left(j\right),l)=0$.

BGLL is an iterative two-phase algorithm based on weighted modularity (WQ) optimization. In the first phase, all nodes are placed into different communities. For each node *i*, BGLL considers each neighbor *j* and evaluates the gain of WQ that would take place if *i* was removed from its community and placed in the community of *j*. Node *i* is then placed in the community for which this gain is maximum and positive. The second phase consists of building a new network whose nodes are now the communities found during the previous phase, and the weights of the edges between the new nodes are given by the sum of the weight of the edges between nodes in the corresponding two communities.

(11) $$WQ=\frac{1}{m^{w}}\sum_{i,j\in V}\left[W\left(i,j\right)-\frac{d_{i}^{w}d_{j}^{w}}{m^{w}}\right]\times \varphi \left(c_{i},c_{j}\right)$$

$m^{w}=\sum_{i,j\in V}W\left(i,j\right)$, $d_{i}^{w}=\sum_{j\in \Gamma_{i}}W\left(i,j\right)$, $c_{i}$ and $c_{j}$ respectively denote the community to which nodes *i* and *j* belong. If $c_{i}=c_{j}$, then $\varphi (c_{i},c_{j})=1$, else $\varphi (c_{i},c_{j})=0$.

*K*-medoids is a clustering algorithm related to the *K*-means algorithm [30]. Its inputs are the similarity matrix and the number of clusters *K*. In our experiments, *K* is set to the true number of clusters. The similarity between two connected nodes is equal to the edge weight that connects them, and the similarity of two disconnected nodes is 0. First, it selects *K* initial medoids randomly; clusters are then defined as the subsets of points that are similar to the respective medoids, and the objective function is defined as the similarity between a point and the corresponding medoid. The new medoids are then updated as the object of a cluster whose average similarity to all the objects in the cluster is maximal. This process is repeated until all medoids no longer change.

## 4. Experiments and Analysis

**Similarity measures for comparison.** This section compares CNS with several representative node similarity measures including Adjacency, Cosine, Jaccard, SMC, and CAS in terms of community detection performance. Table 2 shows the main formulas.

**Baseline methods.** SLPA, BGLL, and *K*-medoids are used. Since SLPA and *K*-medoids are not stable, they are repeated 100 times and averaged for the final results. The value of parameter $\alpha_{n}$ in CAS and CNS is 1/M. *M* is the total number of node attributes. We apply the algorithms on both synthetic and real networks to test their community detection performance.

**Synthetic networks.** The structure-only networks consisting of nodes, edges, and communities are generated according to the LFR benchmark networks [31], which are currently the most commonly used synthetic networks in community detection. An LFR network includes the following parameters: *N* is the number of nodes; avgk is the average degree of the nodes; maxk is the maximum degree of the nodes; minc is the number of nodes contained by the minimum community; maxc is the number of nodes contained by the biggest community; mu is a mixed parameter, which is the probability of nodes connected to nodes of an external community. The greater mu is, the more difficult it is to detect the community structure.

In real networks, not all node attributes are the same important for community detection. Some are critical for cluster nodes, and some are not as important or are not even relevant. Therefore, three kinds of value distributions are generated as follows. (1) Attribute 1: For each community, all of the nodes in a community are assigned the same domain value; (2) Attribute 2: All of the nodes in the network are assigned a random domain value; (3) Attribute 3: All of the nodes in each community are assigned the same domain value. Nodes in the community are selected to host the noise. The noise is a random domain value that is different from the cluster domain value. The noise level nl (the percentage of noise nodes) can be varied.

**Real networks.** Experiments are also conducted on three well-known real networks: the lawyer friendship network (Lazega) [32], the researcher relationship network (Research) [33], and the counselor relationship network (Consult) [33]. The detailed information of each network is shown in Table 3.

Lazega reflects corporate law partnership in a Northeastern US corporate law firm from 1988 to 1991. It includes friendship and working networks between the 71 attorneys of this firm. Various number of attributes are used in this paper, including status (1: partner; 2: associate), gender (1: man; 2: woman), office (1: Boston; 2: Hartford; 3: Providence), years with the firm, age, practice (1: litigation; 2: corporate), and law school (1: harvard, yale; 2: ucon; 3: other).

Research is about a research team consisting of 77 employees in a manufacturing company. The dataset contains several attributes of each employee: location (1: Paris; 2: Frankfurt; 3: Warsaw; 4: Geneva), tenure (1: 1–12 months; 2: 13–36 months; 3: 37–60 months; 4: 61+ months), and the organizational level (1: Global Dept Manager; 2: Local Dept Manager; 3: Project Leader; 4: Researcher). Since the network is a weighted and directed network, we first convert it to an unweighted and undirected network.

Consult is the relationship between 46 employees in a consulting company. The following attributes are known for the counselors: the organisational level (1: Research Assistant; 2: Junior Consultant; 3: Senior Consultant; 4: Managing Consultant; 5: Partner), gender (1: male; 2: female), region (1: Europe; 2: USA), and location (1: Boston; 2: London; 3: Paris; 4: Rome; 5: Madrid; 6: Oslo; 7: Copenhagen). This network is also a weighted and directed network, we first convert it to an unweighted and undirected network.

**Evaluation Criteria.** For networks with known community structure, we use normalized mutual information (NMI) [34], F-Measure [35] and Accuracy as the evaluation criteria to compare results of different algorithms. The calculation formulas are shown as follows.

(12) $$\begin{array}{cc} NMI= & \frac{-2\times \sum_{r=1}^{R}\sum_{k=1}^{K}\frac{|U_{r}⋂C_{k}|}{\left|V\right|}\log\left(\frac{\left|V|\times \right|U_{r}⋂C_{k}|}{|U_{r}\left|\times \right|C_{k}|}\right)}{\sum_{r=1}^{R}\frac{|U_{r}|}{\left|V\right|}\log\left(\frac{|U_{r}|}{\left|V\right|}\right)+\sum_{k=1}^{K}\frac{|C_{k}|}{\left|V\right|}\log\left(\frac{|C_{k}|}{\left|V\right|}\right)} \end{array}.$$

$C=\{C_{1},C_{2},\cdots ,C_{K}\}$ represents a community detection result generated by the evaluated algorithm, and $U=\{U_{1},U_{2},\cdots ,U_{R}\}$ represents the ground-truth community structure. |V| represents the number of nodes in the network. *K* and *R* are the number of communities.

(13) $$F-Measure=\sum_{r=1}^{R}\frac{|U_{r}|}{\left|V\right|}\max_{C_{k}\in C}F\left(U_{r},C_{k}\right).$$

(14) $$F\left(U_{r},C_{k}\right)=\frac{2\times P\left(U_{r},C_{k}\right)\times R\left(U_{r},C_{k}\right)}{P\left(U_{r},C_{k}\right)+R\left(U_{r},C_{k}\right)}.$$

$P\left(U_{r},C_{k}\right)=|U_{r}⋂C_{k}\left|/\right|C_{k}|$, and $R\left(U_{r},C_{k}\right)=|U_{r}⋂C_{k}\left|/\right|U_{r}|$.

(15) Accuracy=TC/|V|.

TC represents the number of correct clustering nodes.

### 4.1. Detection Performance with vs. without Node Attribute Information

This section performs experiments to compare the results of three algorithms based on different similarity methods that do or do not involve node attribute information. The results are shown in Table 4, Table 5 and Table 6. Numbers in bold style means they are the biggest among six similarities.

Table 4, Table 5 and Table 6 show that community detection based on CNS achieves better NMI (e.g., maximally 35.45% improvement on the Consult data), F-Measure (e.g., maximally 12.14% improvement on the Consult data), and accuracy (e.g., maximally 15.14% improvement on the Consult data) when compared with the best result of other structure and attribute similarity measures. The results based on SMC are not always better than those based on structure similarities. This illustrates the importance of considering the complex hierarchical interactions within and between node attributes and network structure when calculating node similarity. When the similarity based solely on the node attribute is compared, CAS cannot guarantee better results than SMC. This means the interactions between node attributes and network structure play a vital role in capturing node similarity.

### 4.2. Effect of Differently Integrating Node Similarities

There are different ways to integrate the proposed node similarity components to form the coupled node similarity. Four combinations are used to obtain CNS: $CNS1=\delta_{m}^{Ia}\times \delta_{m}^{Ie}\times \delta_{m}^{AS}$, $CNS2=\left(\delta_{m}^{Ia}+\delta_{m}^{Ie}\right)\times \delta_{m}^{AS}$, $CNS3=\delta_{m}^{Ia}\times \delta_{m}^{Ie}+\delta_{m}^{AS}$, and $CNS4=\delta_{m}^{Ia}+\delta_{m}^{Ie}+\delta_{m}^{AS}$. These CNSs are then fed into SLPA, BGLL, and *K*-medoids for community detection.

**Figure 3:**
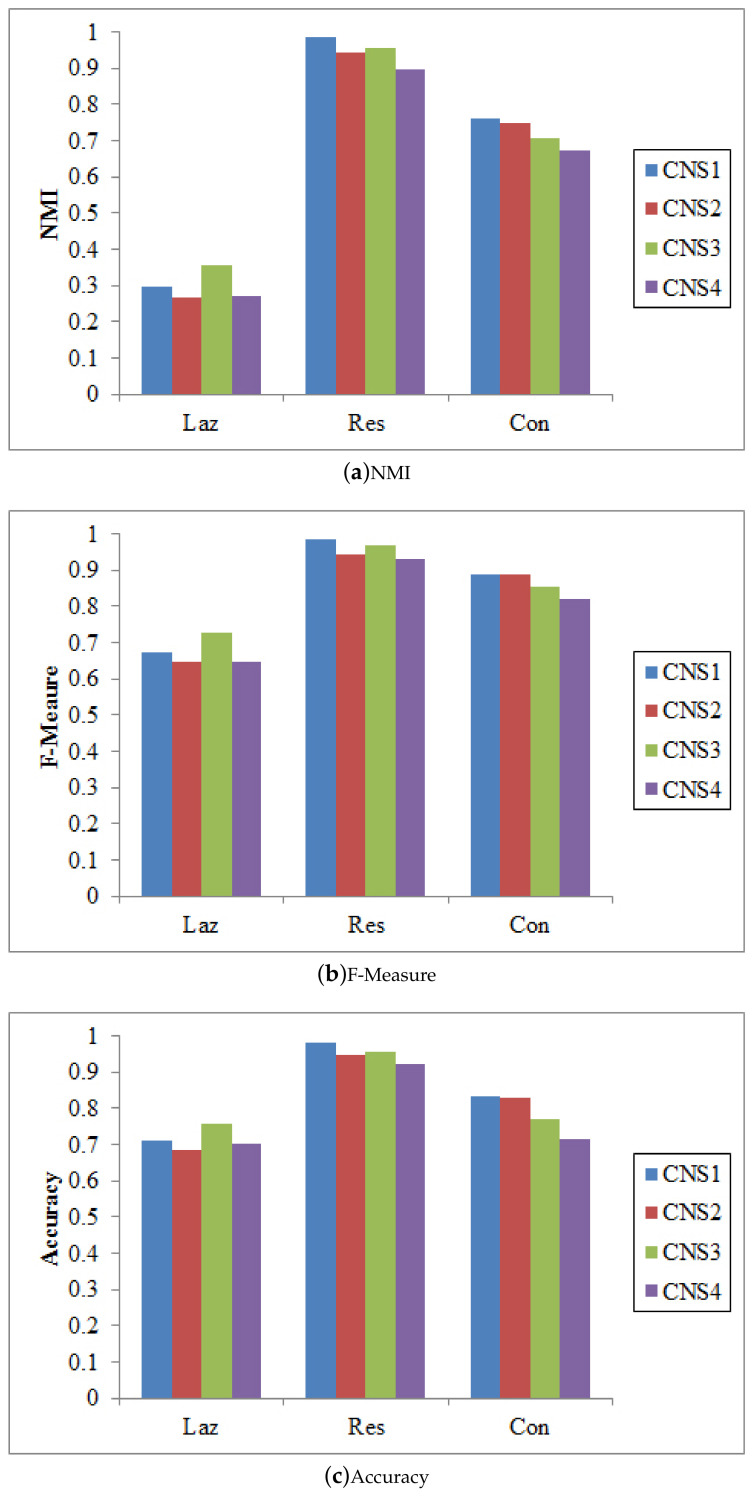
The results of SLPA w.r.t. different CNSs.

**Figure 4:**
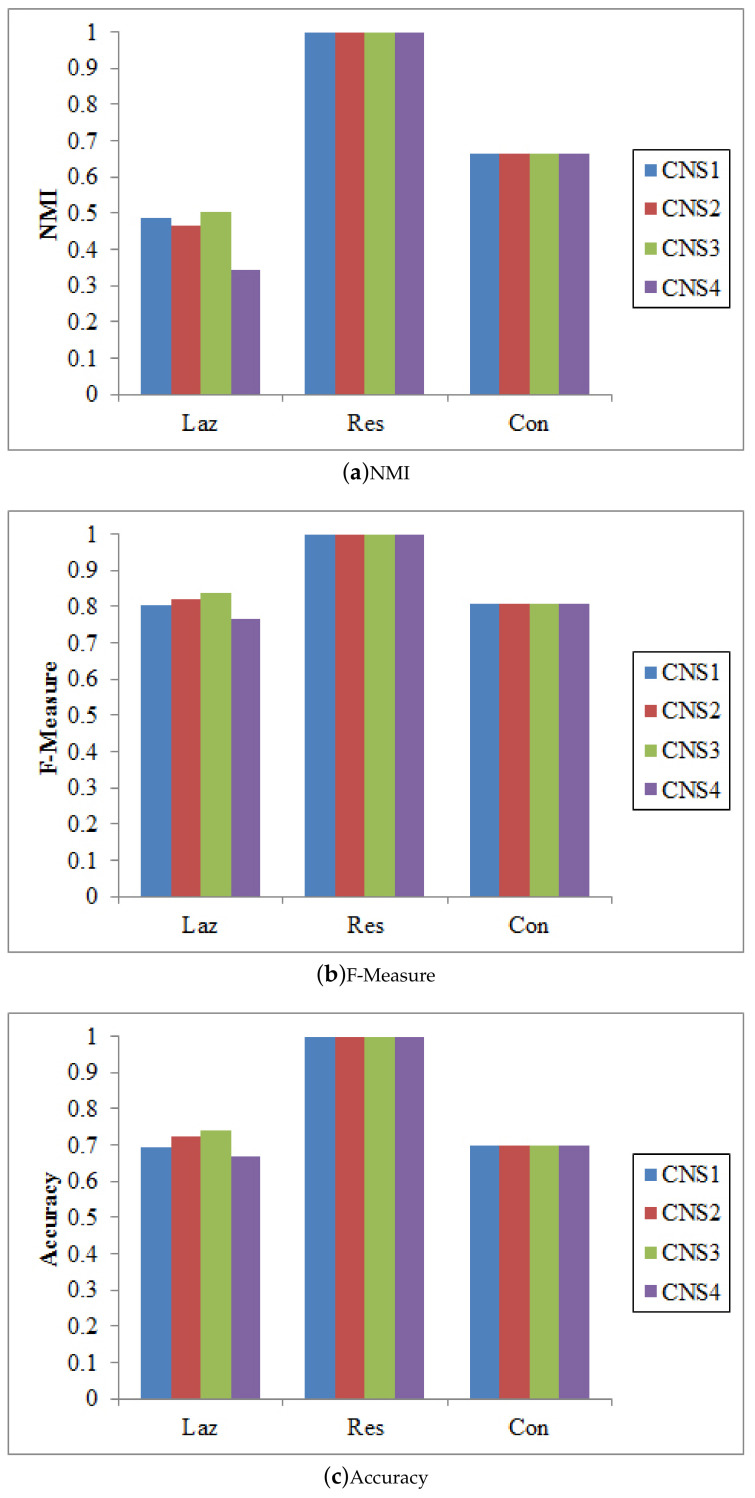
The results of BGLL w.r.t. different CNSs.

**Figure 5:**
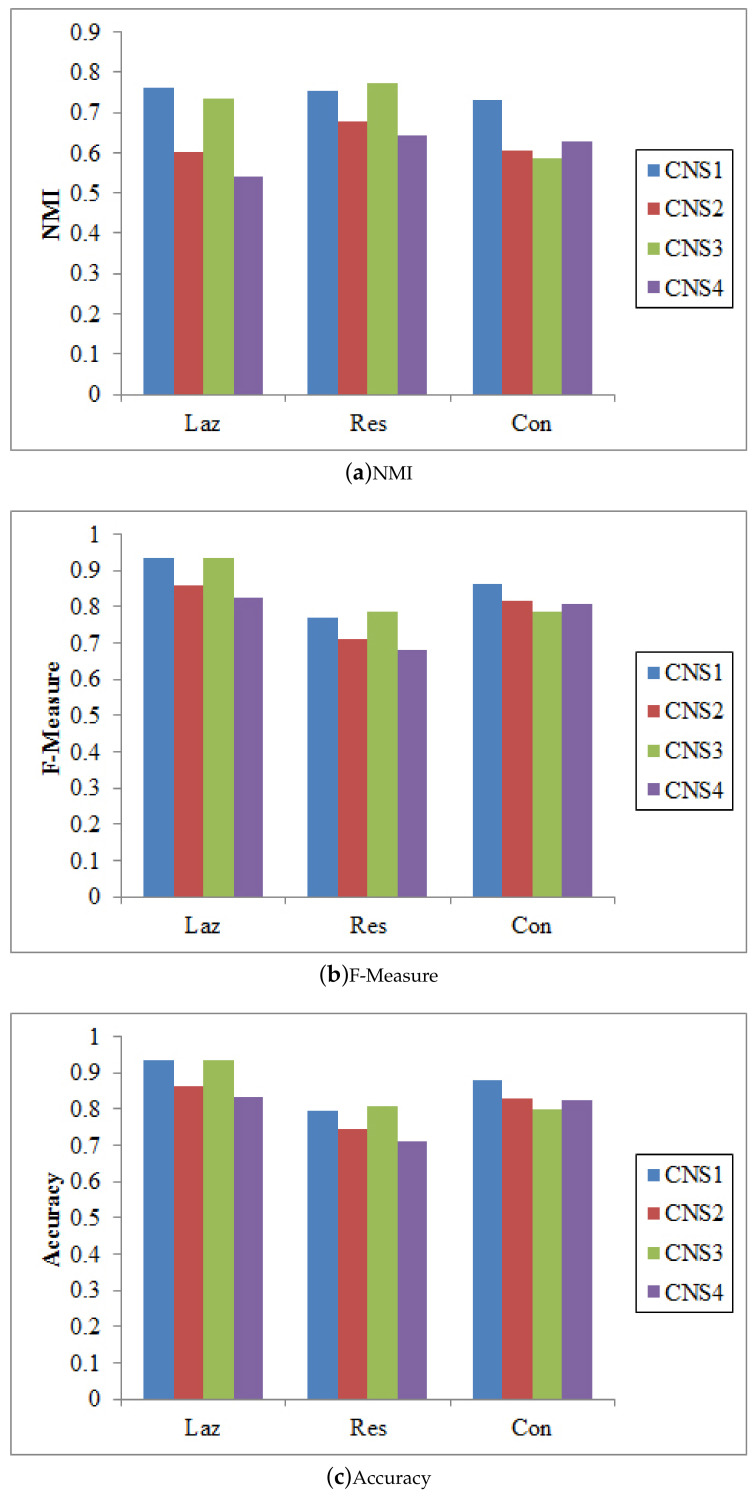
The results of Kmedoids w.r.t. different CNSs.

Figure 3, Figure 4 and Figure 5 show that CNS1 and CNS3 are better in most cases, e.g., CNS1 gains 42.40% improvement of NMI over CNS4 on the Lazega data, and CNS3 gains 47.41% improvement over CNS4. However, we cannot tell which works the best in all cases. Various combinations of the three types of similarities may lead to different results and sometimes the difference is significant (e.g., NMI between 54.21% and 76.02% on the Lazega data). This will be further explored in our future work.

### 4.3. Impact of Varying Network Structure Complexity

We generate nine LFR benchmark networks with N=100, avgk=5, maxk=10, minc=10, and maxc=30, but mu ranging from 0.1 to 0.9 to form networks with different structure complexities. Three attributes (Attributes 1, 2 and 3) are generated for these LFR networks according to the rules of synthetic node attributes and the noise level nl=0.3.

**Figure 6:**
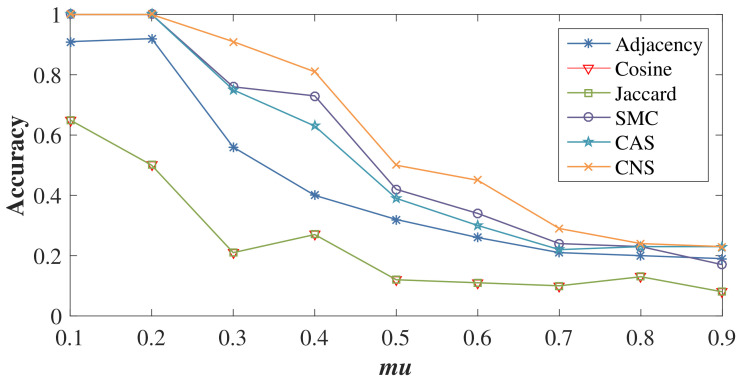
The results of BGLL w.r.t. different levels of network structure complexity.

Figure 6 reports the accuracy of the community detection results using BGLL on these networks. With the increase of mu, the level of separation between the communities decreases and the task of community detection is more difficult. Therefore, the accuracy of all methods decreases. However, CNS-based BGLL achieves better results than other similarity methods. Even when mu=0.9, considering the complex interactions between node attributes and network structure still plays a positive role in the community detection process. BGLL based on Cosine and Jaccard similarity obtain almost the same results, with their two lines overlapping and are also the worst on all nine LFR networks. This verifies that simply considering common neighbors cannot accurately reveal their similarity.

### 4.4. Comparison Against Other Methods

**Figure 7:**
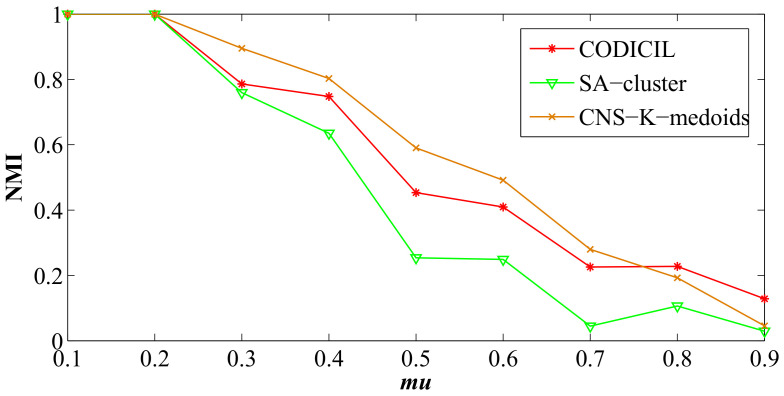
The results of NMI (%) w.r.t. three community detection algorithms on attributed networks.

We generate nine LFR benchmark networks with N=5000, avgk=5, maxk=10, minc=10, and maxc=30, but mu ranging from 0.1 to 0.9 to form networks with different structure complexities. Three attributes (Attributes 1, 2 and 3) are generated for these LFR networks according to the rules of synthetic node attributes and the noise level nl=0.3. We compare the results of CNS-based K-medoids with two other community detection algorithms on attributed networks, e.g., SA-cluster and CODICIL. The results are shown as Figure 7.

From Figure 7, it is observed that the NMI of experimental results on nine different networks decreases with the increasing of parameter mu and the results of the proposed algorithm are optimal in most cases.

## 5. Conclusions

A novel coupled node similarity (CNS) measure is proposed to capture both explicit and implicit interactions between nodes using network structure and node attribute information in complex networks. Different levels of couplings in categorically attributed networks are learned, from node attribute values to nodes and between node attributes and network structure. Empirical analysis verifies the effectiveness of CNS-based community detection in beating several benchmark similarity methods, and, involving different node interactions and handling different levels of network structure complexity, highlights its strengths in terms of whether or not node attributes are involved. However, at present, our proposed method mainly concentrates on unweighted graphs. In the future, we will give some rules for the combination of the new and pre-existing weights to handle the weighted graphs. Our future work will also focus on using non-IID [36] learning on mixed attributed networks considering the coupling between different types of attributes at the attribute level.

## Figures and Tables

**Table 1:** Notation explanation.

Notation	Description
*M*	The number of node attributes
$\left(F_{m}\right)$	The set of all distinct values on the *m*th attribute
$F_{m}\left(i\right)$	The value of the *m*th attribute for node *i*
*K*	The number of communities
*C*	The communities of the network, $C={⋃}_{k=1}^{K}C_{k}$
$c_{i}$	The community to which node *i* belongs
$\Gamma_{i}$	The neighbor set of node *i*
A(i,j)	The adjacency relationship between nodes *i* and *j*. A(i,j)=1 if nodes *i* and *j* are connected; otherwise A(i,j)=0
W(i,j)	The weight between nodes *i* and *j*
S(i,j)	The similarity between nodes *i* and *j*
$l_{r}\left(i\right)$	The received label of node *i*
$m^{w}$	The sum of all edge weights in the network, $m^{w}=\sum_{i,j\in V}W\left(i,j\right)$
$d_{i}^{w}$	The sum of edge weights which are connected to node *i*, $d_{i}^{w}=\sum_{j\in \Gamma_{i}}W\left(i,j\right)$
$g_{m}\left(x\right)$	The node set whose *m*th attribute value is *x*
$\alpha_{n}$	The weight parameter for the *n*th attribute, $\sum_{n=1}^{M}\alpha_{n}=1$, $\alpha_{n}\in \left[0,1\right]$
$\delta_{m|n}\left(x,y\right)$	the inter-relative attribute coupled similarities between values *x* and *y* of the *m*th attribute based on the *n*th attribute (n≠m)
$\bar{B}$	$\bar{B}=F_{n}/B$, the complement set of *B* under the complete distinct value set $F_{n}$ of the *n*th attribute
$g_{n}^{*}\left(B\right)$	the node set whose attribute value in the *n*th attribute is in *B*
$\delta_{m}^{Ia}\left(x,y\right)$	The intra-attribute coupled similarity between the attribute values *x* and *y* of the *m*th attribute
$\delta_{m}^{Ie}\left(x,y\right)$	The inter-attribute coupled similarity between the attribute values *x* and *y* of the *m*th attribute based on other attributes
$\delta_{m}^{A}\left(x,y\right)$	The coupled attribute similarity between the attribute values *x* and *y* of the *m*th attribute
$\delta_{m}^{AS}\left(x,y\right)$	The coupled attribute-to-structure similarity between the attribute values *x* and *y* of the *m*th attribute
CAS(i,j)	The coupled attribute similarity between nodes *i* and *j*
CNS(i,j)	The coupled node similarity between nodes *i* and *j*

**Table 2:** The similarities.

Similarity	Formula
Adjacency	$S_{Adjacency}\left(i,j\right)=A\left(i,j\right)$
Cosine	$S_{Cosine}\left(i,j\right)=\frac{|\Gamma_{i}\cap \Gamma_{j}|}{\sqrt{|\Gamma_{i}\left|\times \right|\Gamma_{j}|}}$
Jaccard	$S_{Jaccard}\left(i,j\right)=\frac{|\Gamma_{i}\cap \Gamma_{j}|}{|\Gamma_{i}\cup \Gamma_{j}|}$
SMC	$S_{SMC}\left(i,j\right)=\frac{\sum_{m=1}^{M}1\left(F_{m}\left(i\right)=F_{m}\left(j\right)\right)}{M}$
CAS	Equation (6)
CNS	Equation (8)

**Table 3:** The information of real networks.

ID	Name	Abbr.	|V|	|E|	*K*	*M*
R1	Lazega	Laz	71	575	2	7
R2	Research	Res	77	2228	3	4
R3	Consult	Con	46	879	4	2

|V|:The number of nodes; |E|: The number of edges; *K*: The number of communities; *M*: The number of node attributes.

**Table 4:** The results of NMI(%) w.r.t. six similarities.

Similarity	SLPA			BGLL			*K*-Medoids		
	Laz	Res	Con	Laz	Res	Con	Laz	Res	Con
Adjacency	14.04	65.68	58.15	31.47	70.92	**66.42**	11.25	29.02	20.15
Cosine	25.81	80.22	64.94	36.65	75.66	49.60	22.61	58.64	37.79
Jaccard	26.16	78.48	64.66	39.13	75.62	49.60	39.76	62.88	30.80
SMC	27.67	92.05	70.46	39.04	**100**	**66.42**	28.46	34.75	47.74
CAS	26.11	87.84	67.23	36.18	86.64	**66.42**	72.55	34.08	54.11
CNS	**29.47**	**98.71**	**78.67**	**48.67**	**100**	**66.42**	**76.02**	**76.46**	**73.29**
Δ%	6.51	7.24	11.65	24.38	0.00	0.00	4.78	21.60	35.45

**Table 5:** The results of F-Measure(%) w.r.t. six similarities.

Similarity	SLPA			BGLL			*K*-Medoids		
	Laz	Res	Con	Laz	Res	Con	Laz	Res	Con
Adjacency	52.32	68.25	80.00	71.96	62.98	**80.87**	53.54	40.36	63.36
Cosine	66.27	87.22	79.81	75.85	89.52	36.67	71.57	70.58	76.66
Jaccard	65.32	85.00	79.62	70.14	86.47	36.67	75.21	74.85	74.52
SMC	65.57	93.89	74.11	73.81	**100**	**80.87**	74.48	54.06	74.48
CAS	64.84	92.60	82.12	77.39	71.55	**80.87**	91.60	52.40	75.78
CNS	**67.38**	**98.93**	**90.79**	**80.41**	**100**	**80.87**	**93.48**	**78.80**	**85.97**
Δ%	1.67	5.37	10.56	3.90	0.00	0.00	2.05	5.28	12.14

**Table 6:** The results of Accuracy(%) w.r.t. six similarities.

Similarity	SLPA			BGLL			*K*-Medoids		
	Laz	Res	Con	Laz	Res	Con	Laz	Res	Con
Adjacency	61.32	73.95	72.60	60.87	66.22	**70.00**	60.14	46.31	67.10
Cosine	63.16	93.47	68.35	63.77	86.49	27.50	72.62	71.55	77.05
Jaccard	59.64	80.35	68.10	55.07	78.38	27.50	76.14	75.97	75.03
SMC	68.22	89.89	74.95	59.42	**100**	**70.00**	75.20	56.92	76.60
CAS	70.26	91.65	71.78	66.67	83.78	**70.00**	92.17	54.03	78.03
CNS	**70.87**	**98.36**	**86.30**	**69.57**	**100**	**70.00**	**93.64**	**80.96**	**88.10**
Δ%	0.87	5.23	15.14	4.35	0.00	0.00	1.59	6.57	12.91
